# Contrast-Enhanced Ultrasonography with Quantitative Analysis allows Differentiation of Renal Tumor Histotypes

**DOI:** 10.1038/srep35081

**Published:** 2016-10-11

**Authors:** Di Sun, Cong Wei, Yi Li, Qijie Lu, Wei Zhang, Bing Hu

**Affiliations:** 1 Department of Ultrasound in Medicine, Shanghai Jiao tong University Affiliated Sixth People’s Hospital, China; 2 Shanghai Institute of Ultrasound in Medicine, China.

## Abstract

Totally 85 patients with 93 renal lesions who underwent contrast-enhanced ultrasound (CEUS) were retrospectively studied with quantitative analysis to evaluate its value in the differential diagnosis of renal tumor histotypes. CEUS characteristics were analysed including the enhancement patterns, peak intensity, homogeneity of enhancement, and pseudocapsule. Quantitative parameters of peak intensity (P) and time to peak (TP) were measured with QontraXt software, and the index “relative enhancement percentage” ΔP% and “difference in TP between tumor and cortex” ΔTP were used to quantify the CEUS features of renal tumors. There are significant difference in CEUS features between the 46 clear cell renal cell carcinoma (CCRCC) and other types of renal tumors, including 17 low malignant lesions, 11 urothelial carcinoma of the renal pelvis, and 19 renal angiomyolipoma. The differences lie in the peak intensity, the homogeneity, the time of wash-in, peak, clearance and presence of pseudocapsule. The ΔTP and ΔP% of the CCRCC is significantly different from other tumors. With “fast to peak + high peak intensity” as the main criterion, assisted with “heterogeneous enhancement” and “fast wash-in” as the secondary criteria, the diagnostic accuracy of CCRCC is 91.4%, demonstrating quantitative CEUS imaging is highly valuable in differentiating CCRCC from other tumors.

Differential diagnosis of renal tumor histotypes is extremely important for clinical treatment decision and prognostic evaluation. Imaging examination is the major basis for differentiation of renal tumor histotypes in clinic and thus is of great significance. About 85% of malignant renal tumors are renal cell carcinomas (RCCs), and in particular, clear-cell RCC (CCRCC) accounts for 70–80%. Meanwhile, papillary RCC (PRCC) and chromophobe RCC (ChRCC), which account for 10–15%, and 5% of all RCCs, are considered as “low-grade malignant renal cell cancers” (LMRCCs), indicating better prognosis than CCRCC. Besides, urothelial carcinoma of the renal pelvis (UCRP) accounts for about 7% of malignant renal tumors^1^. In addition, the most common type of benign renal tumors is renal angiomyolipoma (AML). Among all renal tumors, CCRCC is the most common histotype of malignant renal tumors. Owing to the characteristics of its surface glycoprotein, CCRCC cells are very discrete and more prone to distant metastasis. Thus, CCRCC is more aggressive and demands more positive therapeutic strategies in clinical practice like complete nephrectomy^2^. Whereas LMRCCs and benign tumors are managed conservatively in selected cases. So far, the commonly-used diagnostic methods for renal tumors include routine ultrasound, computed tomography (CT)^3^,^4^, magnetic resonance imaging (MRI)^5^,^6^, histologic test^7^, cytologic test, and molecular test, but none of them is quite satisfactory due to the increasing demand of diagnostic accuracy and less invasiveness in clinical practise. In particular, the existing differential techniques are incapable of quantitative analysis and thus cannot ensure precise and objective assessment.

Thus, the urgent needs in clinic and the increasingly higher expectations from patients for the quality of life have raised the requirements for differential diagnosis of renal tumors. Among all differential diagnosis methods, contrast-enhanced ultrasound (CEUS) imaging technique, which is minimally invasive, real-time, nonradiative, and burdenless on kidney metabolism, is of high reference value for tumor diagnosis, especially for early differentiation, and thus has developed rapidly in recent years. Moreover, the quantitative techniques based on angiograph analysis software are more efficient in reducing subjective errors from observers and contribute to stable, reliable and reproducible results.

According to reports from pathology and molecular biology, CCRCC is featured with rich blood supply, which is different from other renal tumors, including chromophobe RCC, papillary RCC, UCRP and AML, and thus is favorable for efficient preoperative imaging-based differential diagnosis. As reported, papillary RCC and chromophobe RCC are manifested on CEUS as lack of blood supply, and their CEUS parameters (e.g. arrival time, peak intensity, and time of wash-out) are all significantly different from those of CCRCC. UCRPs are mostly characterized by slow wash-in, rapid wash-out, and low peak intensity. Renal AMLs are mostly manifested as slow wash-in and slow wash-out. The accuracy for differentiation of RCCs and renal AMLs based on the criterion of “early clearance, heterogeneous enhancement or pseudocapsule” is 90.5%^8^. Other studies show the accuracy rates from CEUS-based diagnosis of CCRCCs are 48–97%, or 45–82%. However, there is rare report about differentiation with quantitative criteria of CCRCC from other renal tumor histotypes.

In this study, we conducted differential diagnosis with quantitative analysis of CCRCC and other renal tumor histotypes on QontraXt software. The values of CEUS imaging technique for differential diagnosis of various renal tumors was intensively studied and discussed based on quantitative parameters of peak Intensity (P) and time to peak (TP) in CEUS. The quantitative imaging diagnostic criteria for differential diagnosis of renal tumors was also proposed.

## Results

### CEUS with qualitative analysis of 4 renal tumor histotypes

**Figure 1:**
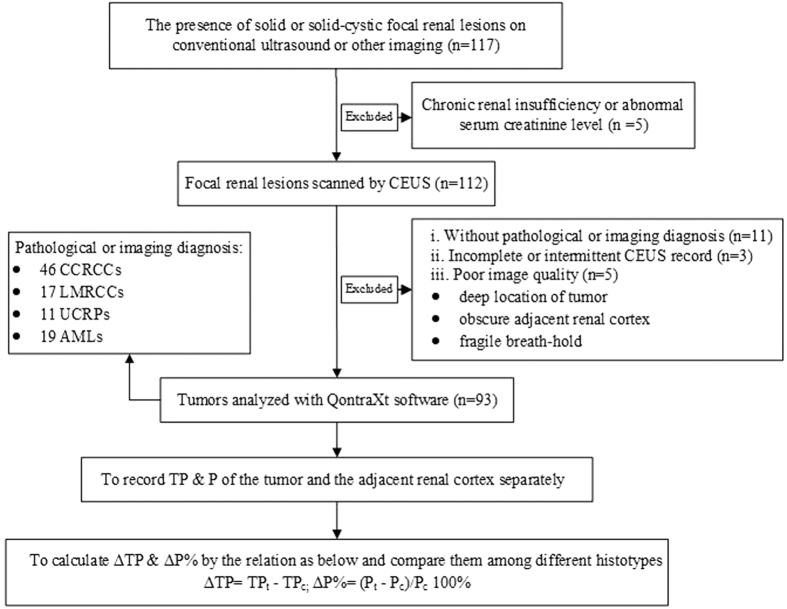
Flowchart of the selection and CEUS analysis of the focal renal lesions. Pt = Peak of the tumor. Pc = Peak of the adjacent renal cortex. TPt = Time to Peak of the tumor. TPc=Time to Peak of the adjacent renal cortex.

The CEUS manifestations of 4 renal tumor histotypes are listed in Table 1. All of the lesions were histopathologically or clinically confirmed. Clearly, six indices (peak intensity, the homogeneity, the time of wash-in, peak, clearance and presence of pseudocapsule) are all significantly different among the 4 histotypes (CCRCC, LMRCC, UCRP, AML) (Fig. 1). In particular, CCRCC is significantly different from other three histotypes in peak intensity, time of wash-in and peak. There are significant statistical difference between LMRCC and CCRCC in the time of wash-in, peak, and wash-out, peak intensity, enhancement homogeneity, and presence of pseudocapsule, with P value of 0.002, <0.001, 0.005, 0.001, <0.001, <0.001, respectively. Meanwhile, statistical differences between UCRP and CCRCC lie in the time of wash-in, peak, peak intensity, and presence of pseudocapsule, with all the P values less than 0.001. Besides, difference is statistically significant between AML and CCRCC in the time of wash-in, peak, and wash-out, peak intensity, enhancement homogeneity, with P value less than 0.001 on the former three parameter and 0.006 on the last one.

### Quantitative characteristics of 4 histotypes on CEUS

The Time-Intensity Curves (TICs) determined from dynamic CEUS images were analyzed on QontraXt for each histotype, and thereby, ΔP% and ΔTP were computed (Table 2). Results show data of ΔTP are significantly different among the 4 histotypes (F = 6.962, P < 0.001), and especially, the ΔTP of LMRCC, UCRP, and AML are all significantly different from CCRCC (p = 0.008, <0.001, 0.019, respectively). Besides, ΔP% are significantly different among the 4 groups (F = 20.02, p < 0.001), and especially, the ΔP% of LMRCC, UCRP, and AML are all significantly different from CCRCC (p < 0.001).

### Differential diagnosis value of CEUS

With “fast to peak + high peak intensity” as the main criterion for diagnosis of CCRCC, assisted with “heterogeneous enhancement” “early wash-in” as references, we found the diagnosis of CCRCC with sensitivity (Sen) 89.4%, specificity (Spe) 93.5%, positive predictive value (PPV) 93.3%, negative predictive value (NPV) 89.6%, and accuracy (Acc) 91.4%.

## Discussion

**Figure 2:**
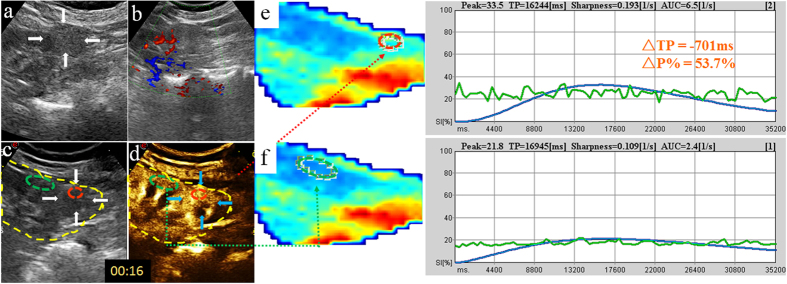
Heterogeneously-enhanced CCRCC in a 79-year-old woman. (**a**,**b**) 2D and CDFI images of the tumor (arrow); (**c**,**d**) Reference scans delimit areas on the CEUS image: the motion compensation area (yellow circle), ROI for analysis area (red circle), ROI for reference area (green circle); (**e**,**f**) Chromatic maps with ROIs (circles) and intensity-time curves supplied by the QontraXt software with ΔTP and ΔP% marked.

**Figure 3:**
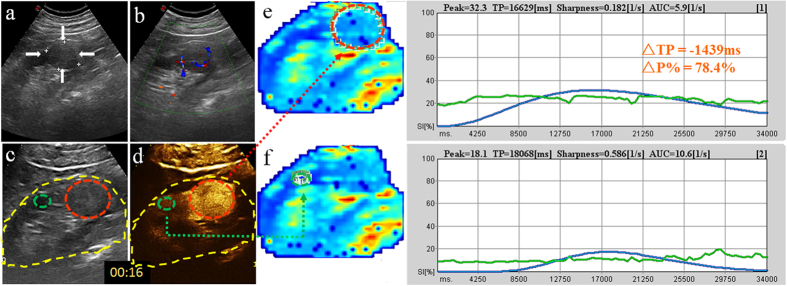
Homogenously-enhanced CCRCC in a 51-year-old man. (**a**,**b**) 2D and CDFI images of the tumor (arrow); (**c**,**d**) Reference scans delimit areas on the CEUS image: the motion compensation area (yellow circle), ROI for analysis area (red circle), ROI for reference area (green circle); (**e**,**f**) Chromatic maps with ROIs (circles) and intensity-time curves created by the QontraXt software with ΔTP and ΔP% marked.

**Figure 4:**
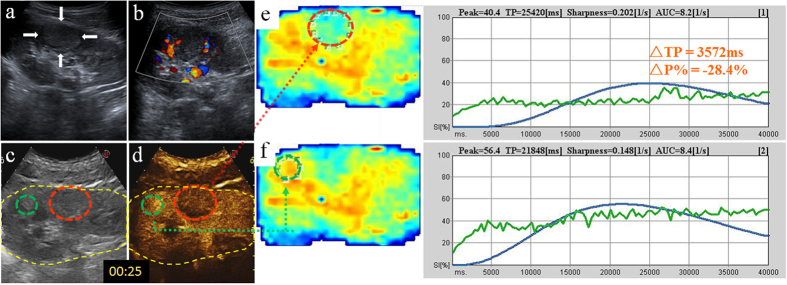
Homogenously-enhanced ChRCC in a 30-year-old woman. (**a**,**b**) 2D and CDFI images of the tumor (arrow); (**c**,**d**) Reference scans delimit areas on the CEUS image: the motion compensation area (yellow circle), ROI for analysis area (red circle), ROI for reference area (green circle); (**e**,**f)** Chromatic maps with ROIs (circles) and intensity-time curves created by the QontraXt software, with ΔTP and ΔP% marked.

Studies conducted on the kidney, liver, thyroid^9^ and other organs^10^,^11^ have demonstrated that CEUS provides a useful, noninvasive and reproducible tool for evaluating the vascularity of lesions. In this study, we qualitatively compared the CEUS characteristics of four renal tumor histotypes (CCRCC, LMRCC, UCRP, AML) on CEUS and found CCRCC is significantly different from other three histotypes on all the six indices (peak intensity, the homogeneity, the time of wash-in, peak, clearance and presence of pseudocapsule) (Fig. 1). In particular, CCRCC is significantly different from other three histotypes in peak intensity, time of wash-in and peak. 80% CCRCCs present as early wash-in, early to peak and high peak intensity on CEUS images compared to the cortex (Figs 2 and 3), whereas other three histotypes like ChRCCs appear as late wash-in, late to peak and low homogeneous enhancement (Fig. 4).The differences are attributed mainly to the pathological characteristics of these tumor types. CCRCC is featured by micro-vessel richness^12^, large and irregular vessels that are distorted, interrupted and densely-grouped, with arteriovenous fistulas^13^, which lead to the rich-supply manifestations of early wash-in, fast to peak, and large peak intensity. CCRCC is mostly manifested as heterogeneous wash-in owing to the rapid tumor growth and proneness to ischemic necrosis. On the contrary, papillary RCC^14^ and chromophobe RCC, UCRP and AML^15^ are all blood-deficient tumors owing to the relative lack of vessels or the thick walls of vessels, so CEUS is able to differentiate CCRCC from them.

CUES imaging is sensitive in differentiating CCRCC from other subtypes of renal tumors. While interpretation of CEUS images depends on the skill and experience of the operator^16^,^17^. Quantitative parametric analysis by Qontraxt allows more objective assessment of CEUS features during the rapid wash-in of contrast^11^,^18^. Parametric imaging is a unique tool analyze the CEUS features of the lesion and the cortex comparatively using predefined parameters like peak intensity and time to peak^6^,^19^. Here, we validated the differences in both time to peak and peak intensity among four histotypes by Qontraxt software. Indices ΔP% and ΔTP were used to reduce the interference from background echo and the arriving time of contrast agent. In particular, the conclusions of peak intensity difference and time to peak are both consistent with the qualitative analysis. We find breathing factors and background echo largely affected the results. Thus, the patients were asked to breathe calmly, and from the respiratory cycles, we selected the areas with stable echo for analysis, which eliminated the interference from respiration.

Finally, we analyzed the qualitative characteristics of CEUS and thereby investigated the diagnostic value for CCRCC. We find with “fast to peak + high peak intensity” as the main criterion, assisted with “heterogeneous enhancement” “early wash-in” as references, the accuracy for CCRCC diagnosis is maximized to 91.4%. Among the misdiagnosed cases, 2 case of chromophobe RCC was manifested on CEUS as “synchronous wash-in, early exit, rapid to peak, high peak intensity”, and was misdiagnosed as CCRCC. One case of papillary RCC was also misdiagnosed as CCRCC, appearing on CEUS images as “early wash-in, synchronous to peak, relatively high peak intensity”. 4 cases of CCRCC was manifested as blood deficiency with “slow to peak, and relatively low peak intensity” and was misdiagnosed as LMRCC. One case of CCRCC protruded to the renal sinus and was manifested as “slow wash-in, slow exit, slow to peak, homogeneous enhancement”, and was misdiagnosed as UCRP. Thus, the combination of clinical manifestations and other imaging characteristics is necessary.

## Conclusions

In conclusion, quantitative analysis assisted CEUS imaging technique was introduced to evaluate the difference of renal tumours, including CCRCC, PRCC, ChRCC, UCRP and AML. The quantitative index “relative enhancement percentage” ΔP% and “difference in TP between tumor and cortex” ΔTP was calculated based on parameters of peak Intensity (P) and time to peak (TP) recorded by QontraXt software, subsequently used to quantify the CEUS features of renal tumors. Using “fast to peak + high peak intensity” as the main criterion, together with “heterogeneous enhancement” and “early wash-in” as references, the accuracy for CCRCC diagnosis is maximized to 91.4%. In particular, CEUS is highly accurate for differential diagnosis of clear cell renal cell carcinoma (CCRCC) from other three common histotypes of renal tumors (LMRCC, UCRP and AML). The results by CEUS imaging technique together with the quantitative analysis of QontraXt software, support the potential of quantitative contrast-enhanced ultrasonography in contributing to the differential diagnosis of renal tumor histotypes in clinical practise.

## Materials and Methods

### Ethics statement

Informed consent was obtained from all patients before their examination. All the experimental protocols of this retrospective research were approved by the local human ethics committee affiliated to Shanghai sixth People’s Hospital. All the methods in this study were performed in accordance with approved guidelines, including the guidelines recommend by European Federation of Societies for Ultrasound in Medicine and Biology.

### Subjects

Following the flowchart of the selection and quantitative analysis of renal tumors on Fig. 1, totally 85 patients with 93 local renal lesions (51 males and 34 females, aged 27 to 86 years, mean 56.5 ± 11.7) who received CEUS in our hospital between Mar. 2012 and May. 2014 were enrolled here. The tumor diameters were 10 to 101 (41.2 ± 21.5) mm. There were 74 malignant lesions (including 46 CCRCCs, 10 chromophobe RCCs, 7 papillary RCCs, 11 UCRPs), and 19 benign lesions which were all AMLs. All malignant lesions and 5 benign lesions were diagnosed via surgical pathology, while other benign lesions were confirmed by two enhanced imaging examinations including CEUS and CT/MRI. All patients denied history of chronic kidney diseases and were normal on renal function examinations including urea nitrogen and creatinine.

### Instruments

We used a Mylab-90 color Doppler ultrasonic instrument (Esaote, Italy) equipped with a real-time CEUS device (CA431 probe, 2.5–5.5 MHz, mechanical index 0.08), and Sonovue (Bracco) ultrasound contrast agents.

### Examination methods

After the optimal sections for observation were selected, it entered the real-time angiograph image-matching mode. The contrast pulse sequence (CPS) was used in the observations. Then via the left elbow vein, 1.2 ml of 11.8 mg/ml Sonovue (prepared in advance) was injected as bolus, and then 5 ml of normal saline was pushed in rapidly. Meanwhile, the built-in timer was started. The patients were asked to respire calmly for real-time observation for 3 min. Dynamic CEUS images were stored in the built-in hard disk for subsequent analysis.

### Image analysis

a) Qualitative analysis.

A senior radiologist retrospectively investigated the dynamic CEUS images. With the adjacent renal cortex at the same depth as a reference, CEUS of the renal tumour was analysed including the time of wash-in, peak, and clearance; enhancement degree, enhancement homogeneity, and presence of pseudocapsule. The enhancement degree of the tumor is classified as hyperenhancement and hypoenhancement, and hyperenhancement means the peak intensity of the tumor is higher or equal to the adjacent cortex.

b) Quantitative analysis of time-intensity curves (TICs).

Digital clips obtained were analyzed with the dedicated software (QontraXt, Italy). First, a region of interest (ROI) was selected manually that encompasses the entire kidney and encoded to the chromatic maps (Figs 2e,f, 3e,f and 4e,f), called “the motion compensation area”. The chromatic maps were composed of a color scale representing different signal intensity (SI), on which red means maximum signal intensity and blue means minimum signal intensity^20^. Then, ROI for analysis area was selected. When a mass showed homogenous enhancement, the entire mass is selected (Fig. 3c,d); when the mass is heterogeneously enhanced, the area with the highest peak intensity was chosen as the ROI (Fig. 2c,d)^21^,^22^. Meanwhile, the adjacent renal cortex at the same depth as the tumor (difference <2 cm) was selected as the ROI for reference area. Next, a Time-Intensity Curve (TIC) was plotted automatically by the system, fitted by the signal intensity changes in the ROIs. Among the quantitative parameters presented on it, peak intensity (P) and time to peak (TP) were recorded. Then, we computed the “tumor relative growth percentage” ΔP% = (Pt − Pc)/Pc × 100% and “difference of time to peak between tumor and cortex” ΔTP = TPt − TPc for further analysis.

### Statistical Analysis

The qualitative indices (time to wash-in, peak, and wash-out; peak intensity, enhancement homogeneity, presence of pseudocapsule) of 4 histotypes of renal tumors were tested via Chi-square test, with significance level at p < 0.05. Paired comparisons were conducted via the adjusted p-values.

Quantitative indices including ΔP% and ΔTP were sent into normality test. Variables in accordance with normal distribution were tested via one-way analysis of variance (ANOVA); paired groups with orderly variance were compared via Student-Newman-Keuls (SNK) test, while paired groups with disorderly variance were compared via Dunnett T3 test. Variables not in accordance with normal distribution were tested via nonparametric rank sum test. P < 0.05 indicates significance.

## Additional Information

**How to cite this article**: Sun, D. *et al.* Contrast-Enhanced Ultrasonography with Quantitative Analysis allows Differentiation of Renal Tumor Histotypes. *Sci. Rep.* **6**, 35081; doi: 10.1038/srep35081 (2016).

## Figures and Tables

**Table 1:** CEUS with qualitative analysis of 4 renal tumor histotypes.

Histotype	No.	CEUS features					
		Early wash-in	Early to Peak	Early wash-out	High peak intensity	Homogeneous perfusion	Pseudo-capsule
**CCRCC**	46	39 (85%)	41 (89%)	26 (56%)	37 (80%)	15 (33%)	27 (59%)
**LMRCC**	17	7 (41%)^a^	7 (41%)^a^	16 (94%)^a^	6 (35%)^a^	15 (88%)^a^	1 (6%)^a^
ChRCC	10	4 (40%)	4 (40%)	9 (90%)	4 (40%)	9 (90%)	1 (10%)
PRCC	7	3 (43%)	3 (43%)	7 (100%)	2 (28%)	6 (86%)	0 (0%)
**UCRP**	11	2 (18%)^b^	2 (18%)^b^	7 (64%)	0 (0%)^b^	8 (73%)	0 (0%)^b^
**AML**	19	4 (21%)^c^	7 (37%)^c^	5 (26%)^d^	2 (10%)^c^	14 (74%)^c^	4 (21%)

^a^*P* < 0.05, LMRCC vs. CCRCC.

^b^*P* < 0.05, UCRP vs. CCRCC.

^c^*P* < 0.05, AML vs. CCRCC.

^d^*P* < 0.05, AML vs. UCRP.

*Data are numbers of cases, with percentage in parentheses.

**Table 2:** Quantitative characteristics of 4 histotypes on CEUS.

Histotype	No. of cases	△TP		△P%	
		Mean ± SD	95% CIs	Mean ± SD	95% CIs
**CCRCC**	46	−1747 ± 2743	−2771~−723	0.324 ± 0.299	0.213~0.436
**LMRCC**	17	2432 ± 2216^a^	382~4492	−0.018 ± 0.072^d^	−0.085~0.049
**UCRP**	11	1065 ± 725^b^	459~1671	−0.124 ± 0.103^e^	−0.211~0.038
**AML**	19	1836 ± 4332^c^	−252~3923	−0.245 ± 0.131^f,g^	−0.332~−0.157

^a,b,c^P < 0.05, LMRCC, UCRP and AML vs. CCRCC.

^d,e,f^P < 0.001, LMRCC, UCRP and AML vs. CCRCC.

^g^P = 0.001, AML vs. LMRCC.

*95% CIs = 95% confidence intervals.
